# Covert dissemination of pLVPK-like virulence plasmid in ST29-K54 Klebsiella pneumoniae: emergence of low virulence phenotype strains

**DOI:** 10.3389/fcimb.2023.1194133

**Published:** 2023-09-27

**Authors:** Jiehui Qiu, Dandan Wei, Jiaxin Ma, Ren Liu, Jianglong Shi, Qun Ren, Chunping Wei, Binghui Huo, Lanlan Zhu, Tianxin Xiang, Yang Liu, Na Cheng

**Affiliations:** ^1^ Department of Infectious Disease, The First Affiliated Hospital of Nanchang University, Nanchang, Jiangxi, China; ^2^ Departments of Clinical Laboratory, Medical Center of Burn Plastic and Wound Repair, The First Affiliated Hospital of Nanchang University, Nanchang, China; ^3^ National Regional Center for Respiratory Medicine, China-Japan Friendship Hospital Jiangxi Hospital, Nanchang, China; ^4^ Department of Pulmonary and Critical Care Medicine, The First Affiliated Hospital of Nanchang University, Nanchang University, Nanchang, China; ^5^ Medical Center for Major Public Health Events in Jiangxi Province, The First Affiliated Hospital of Nanchang University, Nanchang, China; ^6^ Jiangxi Medicine Academy of Nutrition and Health Management, The First Affiliated Hospital of Nanchang University, Nanchang, China

**Keywords:** klebsiella pneumoniae, capsular serotype, ST29-k54, virulence, clinical characteristics

## Abstract

This study aimed to explore the epidemic, clinical characteristics, and molecular and virulence attributes of Klebsiella pneumoniae serotype K54 (K54-Kp). A retrospective study was conducted on 328 strains of Klebsiella pneumoniae screened in a Chinese hospital from January 2016 to December 2019. The virulence genes and antibiotic resistance genes (ARGs) were detected by PCR, and a drug sensitivity test was adopted to detect drug resistance. Multilocus sequence typing (MLST) and PFGE were performed to determine the clonal correlation between isolates. Biofilm formation assay, serum complement-mediated killing, and Galleria mellonella infection were used to characterize the virulence potential. Our results showed that thirty strains of K54-Kp were screened from 328 strains of bacteria, with an annual detection rate of 2.29%. K54-Kp had a high resistance rate to antibiotics commonly used in the clinic, and patients with hepatobiliary diseases were prone to K54-Kp infection. MLST typing showed 10 sequence typing, mainly ST29 (11/30), which concentrated in the B2 cluster. K54-Kp primarily carried virulence genes of aerobactin, silS, allS, wcaG, wabG, and mrkD, among which the terW gene was closely related to ST29 (p<0.05). The strains infected by the bloodstream had strong biofilm formation ability (p<0.05). Most strains were sensitive to serum. Still, the virulence of pLVPK-like virulence plasmid in ST29-K54 Klebsiella pneumoniae was lower than that of ST11 type and NTUH-K2044 in the Galleria mellonella model. Therefore, these findings supply a foundation to roundly comprehend K54-Kp, and clinicians should strengthen supervision and attention.

## Introduction

As the principal cause of hospital-acquired pneumoniae leading to multiple organ failure and even death, Klebsiella pneumoniae (Kp) has become an increasingly common and highly concerned pathogen in recent years (Patel et al., 2014; Russo and Marr, 2019). With the emergence of invasive and metastatic diseases and challenging clinical management caused by multi-drug resistance (MDR), people have gradually realized that Klebsiella pneumoniae with high virulence (HvKP) induces the infection even though MDR occurs based on hypervirulence (Karampatakis et al., 2023). This is a significant threat to patients and a difficult challenge to doctors.

HvKp carries multiple virulence factors, including capsule, lipopolysaccharide, and PLVKP-like virulence plasmid (rmpA, rmpA2, iutA, terW, and silS), resulting in high mortality in mice (Liu et al., 2020; Li et al., 2021). As the main virulence factor of Kp, capsules have been identified as various serotypes. Ten common serotypes belong to HvKp, including K1, K2, K5, K16, K54, K57, and so on, and these strains are characterized by their ability to produce capsular polysaccharide (CPS), supporting bacterial killing and elimination from the human immune system, such as complement and neutrophils (Hsu et al., 2016; Su et al., 2020). The predominant strains of HvKP are K1 and K2, with K57 following closely. Conversely, the occurrence of K54 is infrequently reported worldwide, especially in China. Furthermore, HvKP is usually hypervirulent. K1-Kp can delay the apoptosis of human neutrophils, and K2-Kp has a strong antiserum-killing ability in mouse models with LD_50_ ≤ 10^3^ CFU (Lee et al., 2017; Wang et al., 2021). K57-Kp has a solid antiserum-killing ability (Wei et al., 2021). Turton’s study has indicated that the virulence of the K54-Kp strain ST29 is parallel to that of the ST86 strain, with high virulence belonging to K2-Kp (Turton et al., 2018). Previous studies on K54-Kp are only some case reports (Chuang et al., 2013; Iwasaki et al., 2017). Due to the limited number of cases, a comprehensive understanding of the epidemiology, clinical characteristics, and molecular and virulence features of K54-Kp remains elusive.

Furthermore, our initial research indicated that K54-Kp possesses a remarkable ability to form biofilms, piquing our interest in this study. Hence, through clinical data, drug susceptibility profiles, PCR amplification of virulence and resistance genes, and virulence phenotype experiments, we aim to systematically uncover the clinical and microbial attributes, molecular and virulence characteristics, and the specific molecular traits of this population.

## Materials and methods

### Sample collection and susceptibility identification

A total of 328 Kp strains isolated from the First Affiliated Hospital of Nanchang University in Nanchang City, Jiangxi Province, China, were screened between January 2016 and December 2019. Kp was identified using an automated VITEK-II compact system (bioMerieux, Marcy I’Etoile, France). Thirty of the strains were identified as K54-Kp by polymerase chain reaction (PCR). Retrospectively acquired clinical information on the aforementioned experimental strains of K54-kp comprised demographic information, laboratory indicators, traumatic and primary surgery, treatment with antibiotics, and prognosis. Likewise, medical data on 30 non-K54-KP strains with K54-kP-like sex and age distributions was gathered. The VITEK-2 automated platform (bioMe’rieux, Marcy l’Etoile, France) was used to test the susceptibility of the K. pneumoniae clinical isolates to clinically often used antimicrobial agents in accordance with the manufacturer’s recommendations. Antimicrobial susceptibility tests were conducted using microdilution and were interpreted based on the guidelines provided by the 2020 Clinical and Laboratory Standards Institute (CLSI). The antimicrobials evaluated included ampicillin/sulbactam, piperacillin/tazobactam, cefazolin, ceftazidime, ceftriaxone, cefepime, amikacin, gentamicin, levofloxacin, imipenem, meropenem, cotrimoxazole, tigecycline, and polymyxin(ug/ml,TargetMOI, America).

Salmonella H9812 was detected by pulsed-field gel electrophoresis (PFGE). NTUH-k2044 and ATCC 700603 were used for virulence tests.

### String test

A string test was conducted on a single, freshly-grown colony that had been cultured on an agar plate overnight at 37°C, as described by (Eisenmenger et al., 2021). The inoculation loop was pulled out more than twice. A traction length greater than 5 mm on the sticky loop was considered indicative of a positive hypermucous phenotype (HM).

### Quantification of CPS

The uronic acid-containing CPS content of K54-Kp stains was quantified according to the previously described m-hydroxyphenyl colorimetric method (Mojica and Biofouling, 2010). In simpler terms, a sample from the overnight bacterial culture was taken and resuspended in 0.5 mL of water. This was then vortexed with 1.2 mL of a sodium tetraborate-concentrated sulfuric acid solution in an ice-water bath. The mixture was boiled for 5 minutes, after which 20 µL of 0.15% 3-hydroxybiphenol solution was added. Next, 200 µL of this mixture was transferred to a 96-well plate, and its absorbance was measured at 540 nm. Concurrently, six additional EP tubes were prepared. Each tube received 500 µL of water and glucuronic acid solution. The same procedure that was applied to the bacterial sample was repeated for these tubes to create a standard curve. To determine the polysaccharide content, the OD value of the tested strain was plugged into the formula derived from the standard curve, divided by the strain’s concentration. This measurement was performed in triplicate.

### PCR detection

The DNA of the strains was extracted by boiling method, and the virulence genes and drug resistance genes were amplified by PCR. The following virulence-related genes and antibiotic resistance genes (ARGs) were detected by PCR using the previously designed primers (Guo et al., 2017; Yu et al., 2018; Chen et al., 2022): rmpA, rmpA2, aerobactin, iutA, iucA, iroN,wabG, wcaG, mrkD, allS, silS, and terW, extended-spectrum β-lactamase (ESBL) genes (SHV, CTX-M, and TEM), and carbapenem genes (KPC, NDM, VIM, and OXA-48). The sequences of primers used in this study are summarized in **Table S1**.

### Multilocus sequence typing

MLST typing was conducted on all strains as per the protocol described by Chen et al. (Chen et al., 2022). Seven housekeeping genes of Kp (gapA, infB, mdh, pgi, rpoB, phoE, and tonB) were sourced from the PubMLST website (https://bigsdb.pasteur.fr/klebsiella/) for amplification and sequencing purposes. Alleles and STs were designated using the Kp MLST database (http://bigsdb.web.pasteur.fr/klebsiella/klebsiella.html). The sequences for the housekeeping gene primers are detailed in **Table S2**.

### PFGE

To assess the homology among K54-Kp strains, PFGE was carried out on all strains. Following the protocol described in previous literature, all isolates were digested with XbaI at 37°C for 12 hours (Chen et al., 2022). The PFGE dendrogram was analyzed using NTSYS software to ascertain the relatedness of these isolates. Genetic relatedness was interpreted using a cluster cutoff line set at 85% similarity.

### S1-PFGE and southern blot hybridization

To further investigate whether the virulence and drug-resistance genes were situated on the plasmid and to determine their fragment sizes, we treated the entire chromosomal DNA of the K54-Kp strain with S1 nuclease (Takara, Otsu, Japan) and subsequently transferred the DNA fragments onto a nylon membrane. To ascertain if the two genes were present on the same plasmid, indicating a fusion plasmid, we hybridized with digoxin-labeled rmpA2 and KPC-specific probes. The fragments were then detected using an NBT/BCIP color detection kit (Roche, Mannheim, Germany) as described by Xu et al. (Xu et al., 2019).

### Biofilm formation assay

The crystal violet method was used to measure biofilm at 37°C as previously described (Su et al., 2020), and the absorbance was determined at a wavelength of 540 nm. We used the average value of the broth control group plus three times the standard deviation (x ± 3s) as the negative control value (Ac). The test data were classified explicitly as strongly positive (+++: 4×Ac<A), positive (++: 2×Ac<A ≤ 4×Ac), weakly positive (+: Ac<A ≤ 2×Ac), and negative (-: A≤Ac).

### Serum killing assay

Serum killing assay was performed as described previously (Liu et al., 2017). Briefly, serum was separated from healthy people, packaged, and stored at -80°C. Each strain was tested at least three times. The results were expressed as microbial counts, and the responses regarding viable counts were graded from 1 to 6. A strain was defined as serum sensitive (S) at grades 1–2, intermediately (I) susceptible at grades 3–4, and resistant (R) at grades 5–6.

### Galleria mellonella infection models

The virulence level of the strain was determined by using 300 mg of robust milk yellow Galleria mellonella larvae purchased from Tianjin Huiyu De Biotechnology Company. As mentioned above (Stiel et al., 2021), 3 mL of bacteria cultured in the logarithmic growth phase were centrifuged and precipitated, and 10^8^ CFU/mL of bacterial suspensions were prepared after PBS resuspension precipitation. Next, 10 L (1×10^6^CFU) of the above bacterial suspension was injected into the Galleria mellonella and incubated in an incubator at 37°C for 72 h, and the death was recorded every 12 h. Three parallel experiments were performed. Finally, a survival curve was drawn to show the death of Galleria mellonella larvae.

### Statistical analysis

Statistical analysis was performed using IBM SPSS Statistics Software (ver 24.0) and GraphPad Prism Software (ver 9.0). Data were presented as medians and quartiles or means ± standard deviation. Differences between patient data were analyzed by Chi-square tests or Fisher’s exact test. All tests were two-tailed, and a p-value <0.05 was considered statistically significant.

## Results

### Clinical characteristics and antimicrobial susceptibility of K54-Kp strains

**Figure 1:**
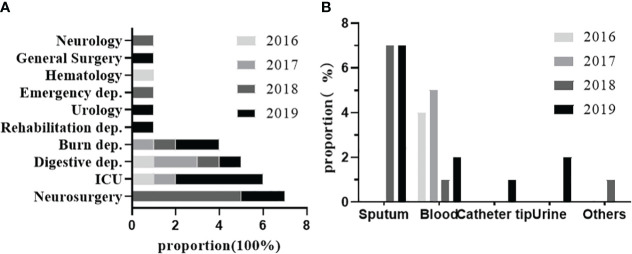
Distribution of strain K54-Kp by department and year **(A)** and by specimen and year **(B)**.

From 2016 to 2019, 30 strains of K54-Kp were detected out of 328 strains of Kp collected in our hospital. The total detection rate was 9.14%, and the average annual detection rate was 2.29%, including 5.8% (4/68)in 2016, 7.6% (5/65) in 2017, 10.8% (9/90) in 2018, and 11.4%(12/105)in 2019. The correlation between the location of clinical departments, the sources of the specimens, and the annual prevalence of K54-Kp is depicted in **Figure 1**. The ICU ward was the primary source of the K54-Kp detection rate’s progressive rise. In addition, we also analyzed the differences in clinical characteristics between K54-Kp patients and non-K54-Kp patients. Upon comparing clinical data, there was no significant difference in general data, invasive procedures, medication, and prognosis between K54-KP and non-K54-KP groups (p>0.05). However, univariate analysis revealed that patients with hepatobiliary diseases were more susceptible to K54-Kp infection (p<0.05) (**Table 1**).

**Table 1:** Comparison of clinical infection characteristics between K54-Kp and non-K54-Kp patients.

Item	K54-Kp n=30	Non-K54-Kp n=30	P
Male (%)	23 (76.70)	24 (80.00)	1.000
Female (%)	7 (23.33)	6 (20.00)	1.000
Stay in ICU (%)	15 (50.00)	22 (73.30)	0.110
Age (year)	60.40±13.14	58.07±14.89	0.522
Hospitalization days before infection (day)	13.87±19.66	11.67±11.26	0.231
Transfer to the department (%)	5 (16.67)	5 (16.67)	1.000
Leukocytosis	15 (50)	16 (53.33)	1.000
Neutrophilia	15 (50)	18 (60)	0.604
Underlying disease			
Chronic pulmonary disease (%)	1 (3.33)	1 (3.33)	1.000
Cardiovascular disease (%)	13 (43.33)	14 (46.67)	1.000
Hepatobiliary diseases (%)	7 (23.30)	0	**0.011**
Chronic nephropathy (%)	1 (3.33)	1 (3.33)	1.000
Diabetes mellitus (%)	7 (23.30)	5 (16.67)	0.748
Nervous system diseases (%)	4 (13.30)	4 (13.30)	1.000
Hematological diseases (%)	0	0	1.000
Tumor diseases (%)	3 (10.00)	0	0.237
Invasive operation			
Mechanical ventilation (%)	13 (43.33)	17 (56.67)	0.439
Tracheal intubation (%)	9 (30.00)	15 (50.00)	0.187
Tracheotomy (%)	10 (33.33)	11 (36.67)	1.000
Central venous catheterization (%)	16 (53.33)	18 (60.00)	0.602
Drainage (%)	18 (60.00)	12 (40.00)	0.121
Nasogastric tube (%)	15 (50.00)	14 (46.67)	0.796
Ureter (%)	24 (80.00)	22 (73.30)	0.542
Operation and traumatic operation (%)	10 (33.33)	12 (40.00)	0.592
Empirical antibiotics received			
Cephalosporin (%)	7 (23.30)	9 (30.00)	0.770
Quinolone (%)	4 (13.30)	3 (10.00)	1.000
Carbapenems (%)	10 (33.33)	14 (46.67)	0.429
β-lactamase inhibitor (%)	16 (53.33)	20 (66.67)	0.429
Aminoglycoside (%)	7 (23.30)	3 (10.00)	0.299
Glycopeptide (%)	3 (10.00)	7 (23.33)	0.299
prognosis (Good %)	14 (46.67)	18 (60.00)	0.301

**Figure 2:**
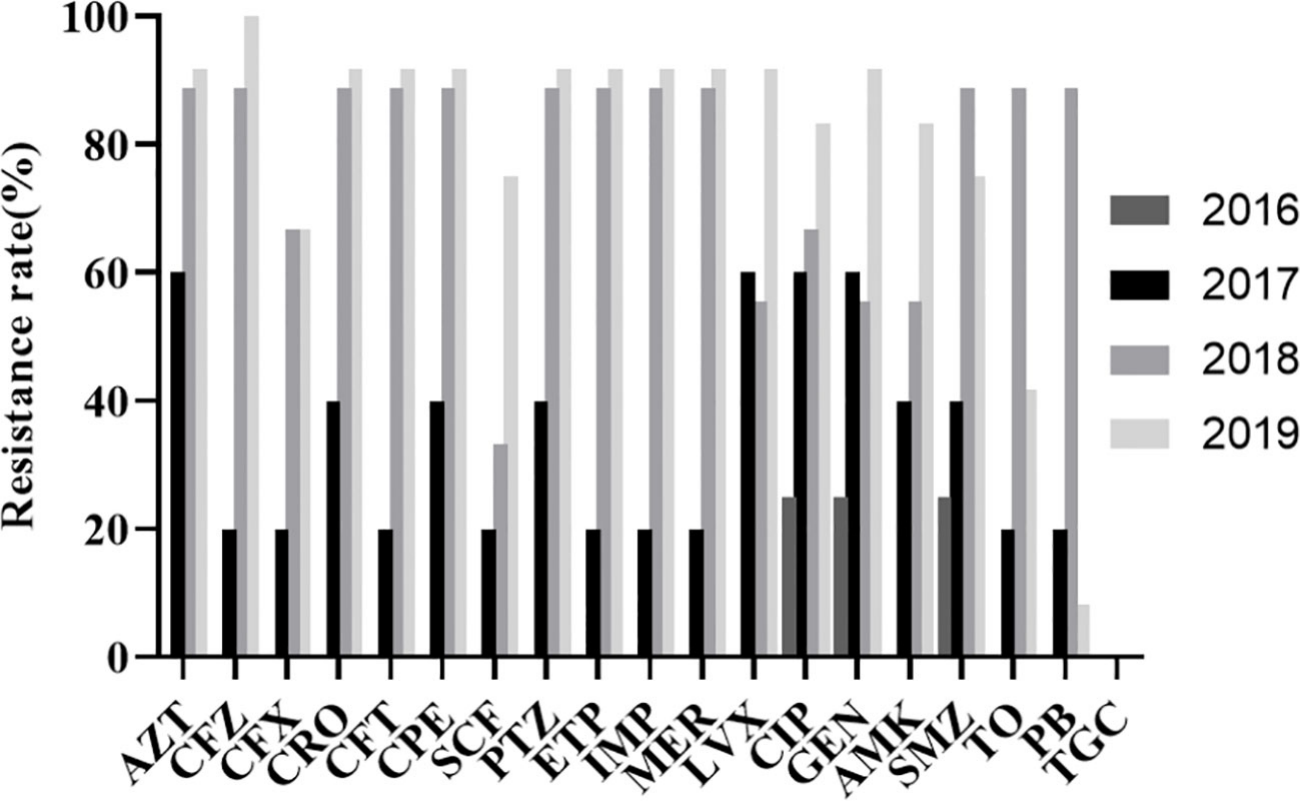
Antibiotic resistance rate of K54-Kp strains.

The resistance rate of K54-Kp to cephalosporins, carbapenems, β-lactamases, and quinolones was increased yearly, with the most significant increase detected in 2018-2019 (**Figure 2**). In contrast, it was susceptible in 2016-2017. The compounds sulfamethoxazole, tobramycin, and polymyxin showed an upward trend in 2016-2018 and decreased significantly in 2018-2019. They were all sensitive to tigecycline in 2016-2019.

### The results of the string test and quantification of CPS

**Figure 3:**
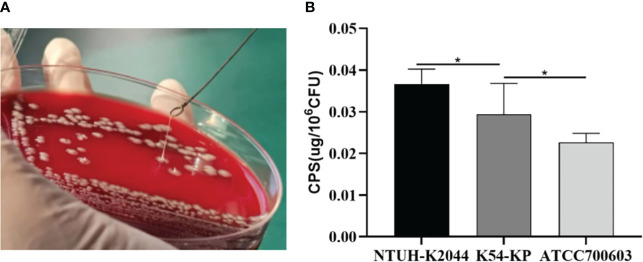
Positive result of a String test **(A)**. Quantification of CPS production of different Kp strains **(B)**. CPS, capsular polysaccharide. *p<0.05.

Analysis revealed that 11 strains (36.67%) tested positive and displayed an HMKP phenotype, with all of them carrying the rmpA/rmpA2 genes (**Figure 3A**). The string test indicated that 36.67% (11 out of 30) of the K54-KP strains tested positive for the HMKP phenotype, and each of these strains contained the rmpA/rmpA2 genes, which are regulators of the mucous phenotype. Notably, some strains possessed the rmpA/rmpA2 gene but did not exhibit the HMKP phenotype. These mucous phenotypic regulatory genes can influence capsule production. As anticipated, the CPS production of K54-KP was significantly lower than that of NTUH-K2044, but it was higher than ATCC700603 (**Figure 3B**). In comparison, there was no significant difference in CPS content between ST29 and non-ST29 strains (p>0.05).

### Epidemiological features

**Figure 4:**
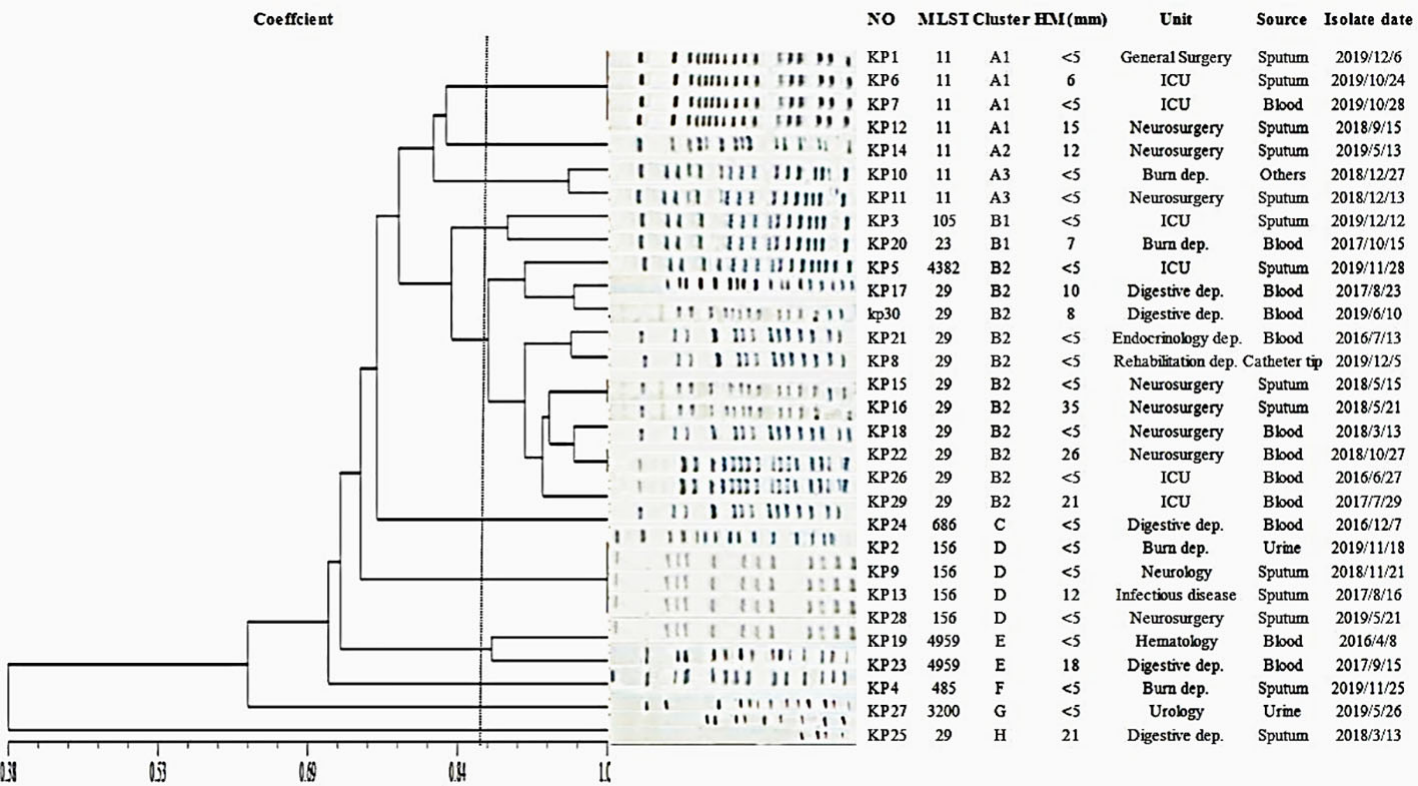
Clonal analysis of k54-Kp strains.

In MLST typing, K54-Kp isolates spread widely. A total of 10 STs were detected, and 36.6% (11/30) of them belonged to ST29, which was the main ST. Other STs included ST11 (7/30, 23.3%), ST156 (4/30, 13.3%), ST4959 (2/30, 6.7%), ST23 (1/30, 3.3%), ST105 (1/30, 3.3%), ST485 (1/30, 3.3%), ST685 (1/30, 3.3%), ST3200 (1/30, 3.3%), and ST4382 (1/30, 3.3%) (**Figure 4**).

The PFGE-based fingerprints of the K54-Kp isolates displayed eight different clusters (named A-H) using a similarity cutoff value of 85% (**Figure 4**), including cluster A (23.3%), cluster B (36.7%), cluster C (3.33%), cluster D (13.3%), cluster E (6.7%), cluster F (3.33%), cluster G (3.33%), and cluster H (3.33%). ST11 strains of K54-Kp were primarily distributed in cluster A, and ST29 stains were in cluster B2.

### Molecular genetic characteristics of K54-Kp strains

**Figure 5:**
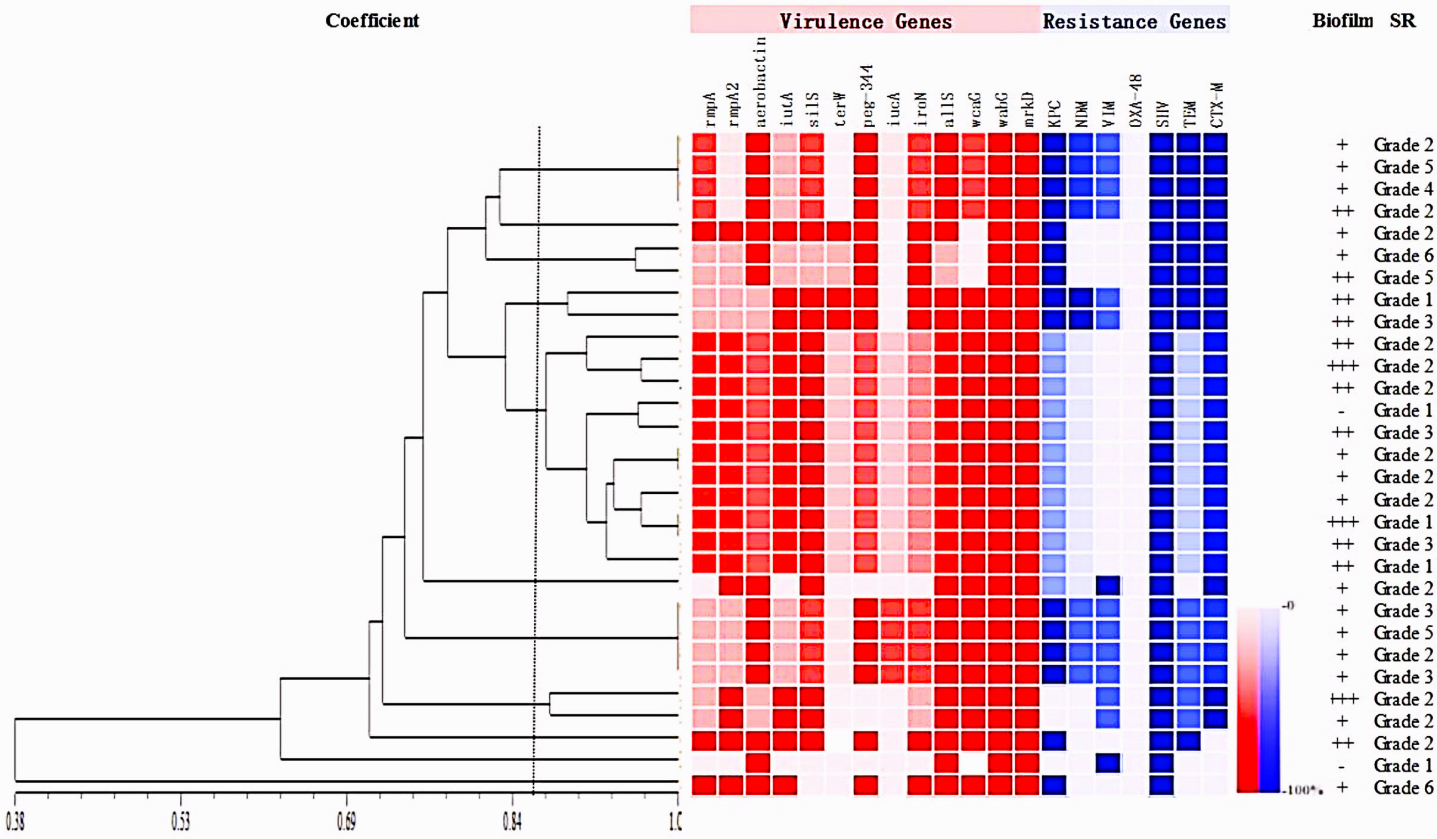
K54-Kp strains virulence, drug resistance gene carrying rate (%), and biofilm and serum resistance results.+++: strongly positive;++: positive; +: weakly positive; -: negative. SR, Serum resistance.

To describe the molecular characteristics of K54-Kp, we examined the distribution of related virulence and drug-resistance genes. In the present study, the expressions of aerobactin, silS, allS, wcaG, wabG, and mrkD genes all exceeded 80%, among which wabG and mrkD genes existed in all strains (**Figure 5**). The proportions of other genes of icuA, iroN, allS, and peg-344 were 36.67%, 66.67%, 90%, and 70%, respectively. Compared with non-ST29 K54-Kp, the terW gene was closely related to ST29 (p<0.05)(**Table 2**). Moreover, the distribution of drug-resistance genes was shown in **Table 3** (Behzadi et al., 2020). Among all the strains, 8strainsharbored the bla_KPC_ and bla_NDM_, and 8strainscarried the rmpA2 and KPC genes.

**Table 2:** Comparison of virulence genes between ST29 and non-ST29 type of K54-Kp.

Virulence gene	K54-Kp(n=30)		P
	ST29(n=11)	non-ST29(n=19)	
rmpA	9	11	0.246
rmpA2	9	9	0.121
aerobactin	9	16	1.000
iutA	10	11	0.100
iucA	5	7	0.712
silS	9	15	1.000
terW	7	3	**0.015**
iroN	6	14	0.425
wcaG	11	15	0.268
peg-344	9	12	0.419
wabG	11	19	–
mrkD	11	19	–
allS	10	17	1.000

**Table 3:** K54-Kp drug resistance gene carrying rate.

Carbapenemase genes		ESBL genes		
blaKPC	20 (66.67%)	blaSHV	30 (100%)
blaNDM	8 (26.67%)	blaTEM	14 (46.7%)
blaVIM	8 (26.67%)	blaCTX-M	24 (80%)
blaOXA-48	0		

### S1-PFGE and southern blot

**Figure 6:**
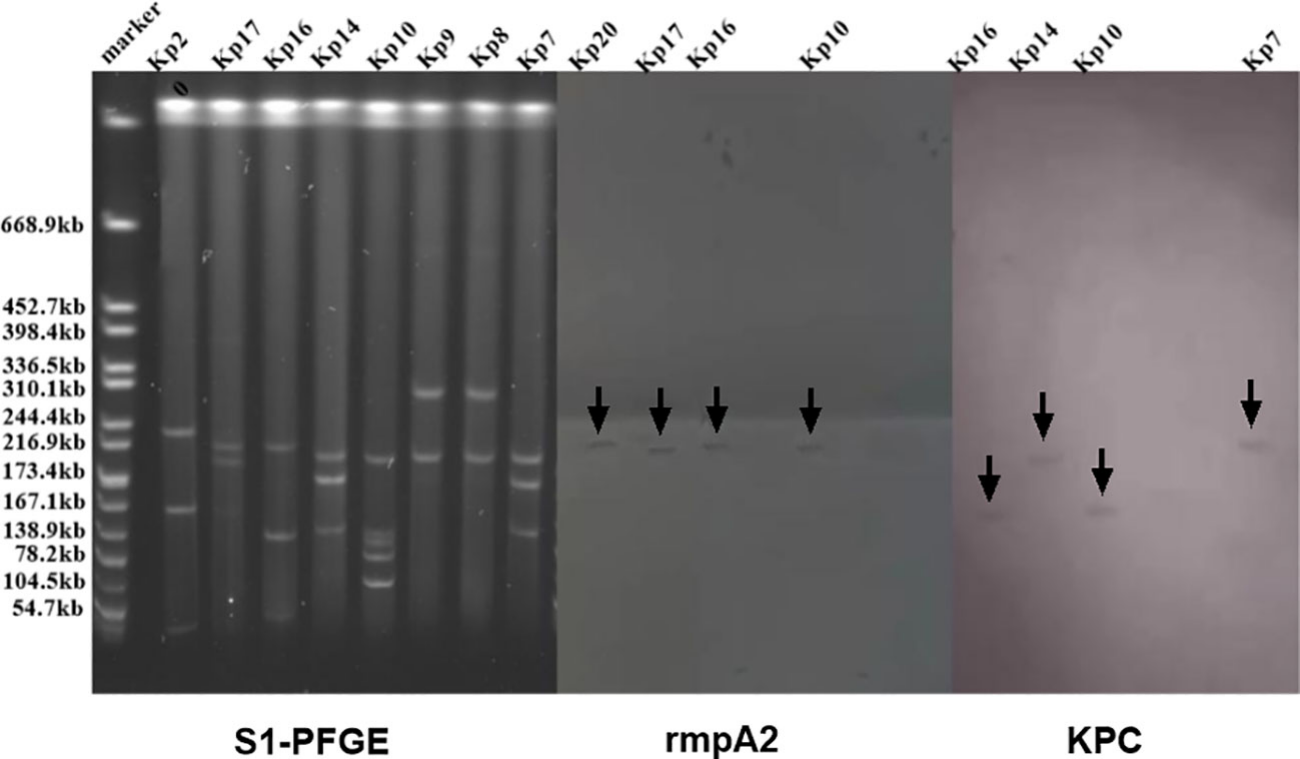
S1-PFGE and Southern blot images of some strains of K54-Kp. Both rmpA2 and KPC genes were detected in the Kp10 and Kp16 strain plasmid.

In prior research, eight strains are identified to carry both the rmpA2 and KPC genes. To ascertain whether these genes were located on plasmids and to determine their fragment sizes, we conducted S1-PFGE and a southern blot, as illustrated in **Figure 6**. The S1-PFGE results demonstrated that all strains harbored between 2 to 4 plasmids of varying sizes, ranging from 54.7 kb to 244.4 kb. The southern blot pinpointed the locations of the rmpA2 and KPC genes within the K54-KP strains. Both the rmpA2 and KPC genes were detected in the Kp16(ST29-K54) and Kp10(ST11-K54) strains. However, they resided on separate plasmids, suggesting that these two strains contained at least two distinct plasmids, rather than a single fusion plasmid.

### Biofilm formation

**Figure 7:**
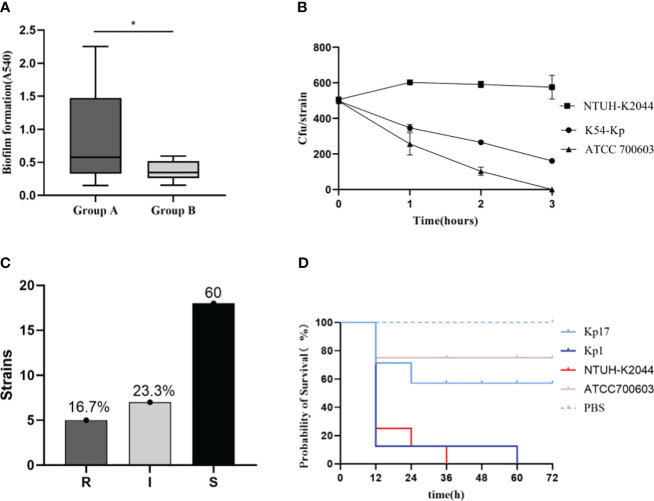
Comparison of biofilm blood flow infection group (group A) and non-blood flow infection group (group B). *p<0.05 **(A)**. Serum resistance test results of K54-KP **(B, C)**. “S” means sensitive, “I” means intermediately, and “R” means resistant. Survival curve of Galleria mellonella infection **(D)**. Kp1 was ST11-K54, Kp17 was ST29-K54.

The biofilm formation ability of the K54-Kp strains differed greatly, but the overall ability was strong. The value of A540 ranged from 0.150 to 2.253, with a median of 0.384. Moreover, 27 (90%) strains could produce biofilm, with an average value of 0.603 ± 0.522 and a negative control range of 0.105-0.279, while three (10%) strains were negative. Among the biofilm-forming strains, three strains (3/27, 11.1%) were strongly positive, 14 strains (14/27, 51.9%) were positive, and 10 strains (10/27, 37.0%) were weakly positive. There was little difference in biofilm formation between ST29 and non-ST29 strains (p>0.05). Interestingly, we found that 90.9%(10/11) of the strains in bloodstream infection samples could form a biofilm, significantly different from those non-bloodstream infections (*p*=0.034) (**Figures 5**, **7A**).

### Serum killing resistance and Galleria mellonella model

We showed the results of serum resistance of K54-Kp (**Figures 5**, **7B, C**). The serum resistance of 30 K54-Kp isolates was between NTUH-K2044 and ATCC700603, which was statistically significant (p<0.05In the Galleria mellonella experiment, strains ST29 and ST11 from the K54-KP group were selected for comparison with NTUH-K2044 and ATCC700603. A survival curve analysis was conducted using these strains alongside PBS, as depicted in **Figure 7D**. Six strains of K54-Kp had the same virulence as NTUH-K2044, and four had lower than ATCC700603.

## Discussion

Kp has attracted much attention recently due to its clinical invasion and drug resistance. It reportedly accounts for 86% of clinical infections, which has become the chief pathogen of healthcare-related infections (Guo et al., 2017; Liao et al., 2022). As a member of HvKP, K54-Kp should have some of the above-related characteristics. Therefore, we aimed to describe its molecular and clinical infection characteristics comprehensively. As expected, K54-Kp was not a dominant group, with a low prevalence rate, which was in line with the results of Liao’s (Liao et al., 2022). Our findings indicated that fewer strains were detected in 2016 and 2017. However, there was a gradual increase in 2018 and 2019, suggesting that the prevalence of K54-KP strains has been rising over the years. This trend indicates that they may become even more widespread in the future. Furthermore, while the main sources of the specimens in 2018 were from the neurosurgical ward and from the ICU ward in 2019, sputum remained the dominant specimen type. In 2018, the strains especially came from sputum in neurosurgery wards, and in 2019, they were primarily from ICU wards, where sputum was still the majority. K1/K2 is deemed to be closely related to community infections(Siu et al., 2012).

In contrast, our research found that K54-Kp was more from nosocomial infection (73.33%). Univariate analysis revealed that patients with hepatobiliary diseases were more susceptible to K54-KP infection. When patients with primary hepatobiliary disorders experience a high fever accompanied by liver pain, it is essential to consider the possibility of a liver abscess caused by K54-Kp as the primary diagnosis. Therefore, clinicians should be vigilant and monitor these patients closely.

The drug sensitivity results revealed a high resistance rate of K54-Kp to common clinical antibiotics. The trend of drug resistance increased significantly over the years, particularly during 2017-2018, and peaked in 2018-2019. Additionally, 66.67% of the strains in this study were classified as MDR-Kp, with CRKP accounting for more than half of these cases. The emergence of MDR-Kp with high drug resistance poses a significant challenge and makes K54-Kp the next “superbacterium,” leading to greater clinical drug resistance and more complicated management(Algammal et al., 2023). Moreover, morbidity and mortality rates are expected to rise considerably.

The string test is a widely used approach to identify strains with HM. In the present study, only 36.7% of strains were positive, while all of them contained the rmpA/rmpA2 gene, which could facilitate the production of CPS and perform a high-viscosity phenotype. However, certain strains that carried the mentioned genes did not exhibit high viscosity characteristics, indicating that solely relying on the string test results is insufficient to determine whether a strain is HvKP (Sheng et al., 2022). Additionally, genes such as iutA, terW, and silS also collectively contribute to the high-viscosity phenotype of HvKp (Li R. et al., 2020). The positive rate of the string test aligned with the results from the quantification of CPS, which is indicative of Kp virulence. When compared to NTUH-K2044, the CPS content of the K54-Kp isolate was found to be lower. This observation was unexpected for us, especially considering that wabG and wcaG, which code for lipopolysaccharide, had high prevalence rates (100% and 86.7%, respectively). These genes can jointly regulate capsule formation with rmpA (Zheng et al., 2018). Much like the rmpA mutant, capsule production is potentially linked to the CPS promoter. Furthermore, removing rmpA results in reduced promoter expression (Cheng et al., 2010), though the specific underlying mechanism requires further investigation.

MLST is a molecular typing tool used to trace the origin of bacteria owing to its good repeatability and high resolution. The well-known K1-Kp is ST23, K2-Kp mainly includes ST65 and ST86, and K57-Kp is ST412(Liu et al., 2021; Nakamura et al., 2021; Shao et al., 2021). At present, domestic and foreign literature reports on K54-Kp are mostly ST29. In our present study, ST29 was still dominant, mainly from blood specimens (7/11), which was rarely reported in the previous literature. Afterward, PFGE results showed that ST29 K54-Kp was concentrated in the B2 cluster, mainly distributed in neurosurgery in 2018, pointing out that there might be a clonal transmission of K5-Kp isolates in our hospital.

Numerous virulence genes, including pLVPK-like virulence genes (rmpA, rmpA2, iucA, iutA, iroN, etc.), occurred in K54-Kp strains. WabG, mrkD, aerobactin, and wcaG were the most, while the terW gene chiefly existed in ST29(*p*=0.015). Moreover, Alka Hasani has also found that the wcaG gene, associated with capsular polysaccharide synthesis, significantly correlates with K54-Kp isolates (*p*= 0.001) (Hasani et al., 2020). K54-Kp also took along diverse drug-resistance genes. The detection of drug-resistance genes agreed with the results of drug sensitivity, which sustains the argument that multiple drug-resistance genes jointly involved the drug-resistance characteristics of bacteria (Su et al., 2020). As a pivotal gene of carbapenem resistance, KPC enzymes were the most prevalent. Remarkably, we did not detect any K54 strains producing the OXA-48 gene. OXA-48, a class D carbapenemase enzyme, is regarded as the predominant carbapenemase gene in hvKp strains in Europe, whereas the KPC gene takes precedence in China (Liapis et al., 2014). The primary Kp strain carrying the OXA-48 gene is identified as the CG101 (ST 101) type in 38 out of 2,298 cases, as cited by Palmieri M et al. (Palmieri et al., 2020). The absence of the blaOXA-48 gene in our findings might be attributed to the geographical distribution trends of OXA-48 (Pitout et al., 2019). Furthermore, Kp strains producing the OXA-48 gene typically exhibit a lower resistance level to drugs compared to those with the KPC and ESBLs genes. However, in China, there’s a higher prevalence of resistance to meropenem and imipenem.

A notable observation was the coexistence of virulence and resistance genes. The hybridization data from S1-PFGE and southern blot revealed distinct molecular weights for the virulent and drug-resistant plasmids in the K54-Kp strains. We sought to discern if the plasmids containing both the rmpA2 and KPC genes were identical. Southern blot results indicated that while these strains housed heterozygous plasmids carrying both virulence and drug resistance genes, no fusion plasmid was present. According to Xie et al. (Xie et al., 2020), such heterozygous plasmids might encode both hazardous and resistant traits by amalgamating structural sections from two separate plasmids. Li et al. (Li D. et al., 2020) have detailed that two homologous regions encoding the mobile element shared by the plasmid and the Group II intron reverse transcriptase can recombine to form the Kp hybrid plasmid.

Biofilms serve as a formidable defense mechanism for sessile bacteria. Strains of K54-Kp involved in bloodstream infections are predisposed to biofilm formation, which results in enhanced virulence. Given that MDR-Kp strains can potentially form biofilms, we delved into their drug susceptibility profiles for deeper insights (James et al., 2019). Our findings revealed that while most strains were susceptible to carbapenem antibiotics, they demonstrated resistance to extended-spectrum β-lactam antibiotics. Notably, there was a significant propensity for ESBLs-Kp to form biofilms in bloodstream infections (P<0.05). The heightened biofilm-forming ability among K54-Kp strains derived from sources causing bloodstream infections not only facilitates the spread of resistance genes among bacteria but also augments the adaptability and survival of K54-Kp in hostile environments. In serum complement-mediated killing experiments, only 16.7% of the strains showed resistance to serum complement, all of which were resistant to carbapenem in sputum. In contrast to MDR bacteria, which typically have great adaptability or low virulence, carbapenem-sensitive strains display no resistance to complement-mediated death (Hennequin and Robin, 2016).

The virulence of ST29-K54Kp was lower than that of ST11 type and NTUH-K2044 in the Galleria mellonella model, contrary to Shao’s research(Shao C. et al., 2022). To further clarify, we analyzed further analysis the virulence genes carried by ST29-K54. The ST29-K54 was discovered to be more dominant in carrying the rmpA2, wcaG, and aerobactin genes (p<0.05), as opposed to the rmpA, iutA, and iroN genes. This finding could indicate that the strain’s gene-environment inhibited or hindered the expression of virulence genes, and the detailed mechanism requires further investigation. Additionally, in the Galleria mellonella model, the strains with positive biofilm development ability and serum resistance did not exhibit correspondingly high pathogenicity, which may also be associated with environmental factors of the strains, as well as the patient’s primary diseases, the number of complications, and the standard antibiotic treatment.

## Conclusion

In conclusion, our study described the overall situation of K54-Kp in a Chinese-affiliated hospital. To our knowledge, this is the first systematic report of K54-Kp. K54-Kp showed a gradually increasing detection rate, highly invasive and pathogenic, and high biofilm formation ability of bloodstream infection strains. Furthermore, the prevalence of MDR-K54 Kp and the development of resistance mechanisms are triggering a global crisis. Therefore, supervision and attention should be strengthened in medical work. However, there are certain restrictions. To begin with, the quantity of samples should be enhanced so as to more extensively and completely depict the K54-KP characteristics. In addition, WGS sequencing need to be conducted with the aim to comprehend the strain’s genomic data when exploring the covert transmission of virulence plasmid in ST29-K54 kp. In-depth research should also be conducted on the mechanism of covert transmission of virulence plasmids, including whether or not it is connected to the expression of the genes contained by these plasmids (detection of plasmid copy number).

## Data availability statement

The original contributions presented in the study are included in the article/**Supplementary Material**. Further inquiries can be directed to the corresponding authors.

## Ethics statement

Ethical approval was not required for the studies on humans in accordance with the local legislation and institutional requirements because only commercially available established cell lines were used. Ethical approval was not required for the studies on animals in accordance with the local legislation and institutional requirements because only commercially available established cell lines were used.

## Author contributions

JQ and DW performed the laboratory measurements and contributed equally to this manuscript. YL and NC made substantial contributions to the design and revised the article critically for important intellectual content. TX was in charge of organizing the supplementary experiment. JQ drafted the article. JM, QR, CW, BH participated in the analysis and interpretation of datas. LZ was responsible for the operation of the supplementary experiment. All authors read and approved the final article.
