# Supplementary material for: Covert dissemination of pLVPK-like virulence plasmid in ST29-K54 Klebsiella pneumoniae: emergence of low virulence phenotype strains
Source: Front Cell Infect Microbiol. 2023 Sep 27;13:1194133. doi: 10.3389/fcimb.2023.1194133 (PMC10565659; doi:10.3389/fcimb.2023.1194133)
Supplement: Supplementary file 1 [file Table_1.docx]

| **Primer name** | **Primer name（5’-3’）** | **Length (bp)** | **References** |
| --- | --- | --- | --- |
| RmpA | F:ACTGGGCTACCTCTGCTTCA  R:CTTGCATGAGCCATCTTTCA | 516 | Virulence genes (Y G. et al., 2017, Chen S. et al., 2022) |
| RmpA2 | F:CTGTGTCCACTATTGGTGGG  R:GATAGTTCACCTCCTCCTCC | 1045 |  |
| wcaG | F:GGTTGGKTCAGCAATCGTA  R:ACTATTCCGCCAACTTTTGC | 169 |  |
| aerobactin | F:GCATAGGCGGATACGAACAT  R:CACAGGGCAATTGCTTACCT | 556 |  |
| allS | F:CCGTTAGGCAATCCAGAC  R:TCTGATTTA(A/T)CCCACATT | 1090 |  |
| iroN | F:AAGTCAAGCAGGGGTTGCCCG  R:GACGCCGACATTAAGACGCAG | 665 |  |
| peg-344 | F:CTTGAAACTATCCCTCCAGTC  R:CCAGCGAAAGAATAACCCC | 425 |  |
| iuc A | F:ATAAGGCAGGCAATCCAG  R:TAACGGCGATAAACCTCG | 189 |  |
| mrkD | F:CCACCAACTATTCCCTCGAA  R:ATGGAACCCACATCGACATT | 240 |  |
| silS | F:CATAGCAAACCTTCCAGGC  R:ATCGGCAGAGAAATTGGC | 803 |  |
| terW | F:ATGCAATTAAACACCAGACAG  R:CTCATTCTCTTGAGTGTTTTC | 239 |  |
| iutA | F:ACCTGGGTTATCGAAAACGC  R:GATGTCATTAGCCTGATTGC | 11115 |  |
| wabG | F:ACCATCGGCCATTTCATAGA  R:CGGACTGGCAGATCCATATC | 683 |  |
| KPC | F:GCTACACCTAGCTCCACCTTC  R:ACAGTGGTTGGTAATCCATGC | 989 | Resistance genes (Yu F. et al., 2018) |
| NDM | F:GGGCAGTCGCTTCCAACGGT  R:GTAGTGCTCAGTGTCGGCAT | 475 |  |
| VIM | F:TCCGACAGTCAGCGAAAT  R:GCAGCACCAGGATAGAAGA | 435 |  |
| OXA-48 | F:CGCATCTTGTTGTCCAAGTG  R:TCGAGCATCAGCATTTTGTC | 1012 |  |
| SHV-1 | F:GCCTTTATCGGCCTTCACTCAAG  R:TTAGCGTTGCCAGTGCTCGATCA | 898 |  |
| TEM-1 | F:TCGGGGAAATGTGCG  R:TGCCTTAATCAGTGAGGCACC | 972 |  |
| CTX-M | F:TACCGCAGATAATACGCAGGTG  R:CAGCGTAGGTTCAGTGCGATCC | 355 |  |

Table S1. Primer sequence of virulence and drug resistance genes
