# Supplementary material for: Covert dissemination of pLVPK-like virulence plasmid in ST29-K54 Klebsiella pneumoniae: emergence of low virulence phenotype strains
Source: Front Cell Infect Microbiol. 2023 Sep 27;13:1194133. doi: 10.3389/fcimb.2023.1194133 (PMC10565659; doi:10.3389/fcimb.2023.1194133)
Supplement: Supplementary file 2 [file Table_2.docx]

Table S2. The sequences for the housekeeping gene primers

| **Primer name** | **Primer name（5’-3’）（5’to 3'）** | **Product length（bp）** | **reference** |
| --- | --- | --- | --- |
| rpoB | F:GGCGAAATGGCWGAGAACCA | 501 | (Chen S. et al., 2022) |
|  | R:GAGTCTTCGAAGTTGTAACC |  |  |
| gapA | F:TGAAATATGACTCCACTCACGG | 450 |  |
|  | R:CTTCAGAAGCGGCTTTGATGGCTT |  |  |
| mdh | F:CCCAACTCGCTTCAGGTTCAG | 477 |  |
|  | R:CCGTTTTTCCCCAGCAGCAG |  |  |
| pgi | F:GAGAAAAACCTGCCTGTACTGCTGGC | 432 |  |
|  | R:CGCGCCACGCTTTATAGCGGTTAAT |  |  |
| phoE | F:ACCTACCGCAACACCGACTTCTTCGG | 420 |  |
|  | R:TGATCAGAACTGGTAGGTGAT |  |  |
| infB | F:CTCGCTGCTGGACTATATTCG | 318 |  |
|  | R:CGCTTTCAGCTCAAGAACTTC |  |  |
| tonB | F:CTTTATACCTCGGTACATCAGGTT | 414 |  |
|  | R:ATTCGCCGGCTGRGCRGAGAG |  |  |
